# Utilizing machine learning for predicting heart failure outcomes: A path toward developing a patient‐centered approach

**DOI:** 10.1002/clc.24260

**Published:** 2024-03-25

**Authors:** Rayyan Nabi, Tabeer Zahid, Hanzala A. Farooqi

**Affiliations:** ^1^ Islamic International Medical College Rawalpindi Pakistan; ^2^ Foundation University Medical College Islamabad Pakistan

To the Editor,

Heart failure (HF)—a global pandemic—poses a huge burden to healthcare systems, with a staggering 64.3 million people worldwide estimated to suffer from the ailment in 2017. Projections suggest a total cost of around $69.8 billion for HF by the year 2030 in the United States.^1^ This highlights the immense economic burden of the disease and calls for effective strategies vis‐à‐vis its treatment and more importantly, prevention. Recent studies have outlined how machine learning (ML) can be used to build predictive models from multidimensional datasets. This has led to the establishment of the role of AI in early detection of future mortality and destabilizing episodes, therefore allowing for the optimization of cardiovascular disease outcomes.^2^

A recent study published by Ketabi et al. analyzed the performance of 10 ML algorithms and chose the best algorithm to predict mortality and readmission of HF patients.^3^ Two thousand four hundred and eighty‐eight patients' information was documented after their first hospital admission and they were then followed to determine three outcomes: hospital readmission, 1‐month mortality, and 1‐year mortality. 14.7% of these patients were readmitted to the hospital, 3.9% died within a month and 13.7% died within a year. To determine this, 57 different factors were considered independent variables to predict outcomes and were entered into and evaluated using ML algorithms. The data were divided into two sets: training sets for the machine algorithm to teach itself, and test sets for evaluating the classifier's prediction error rate after learning. The five metrics utilized to compare the models were accuracy, sensitivity, specificity, F1 score, and AUC. Out of the 10 ML algorithms, CatBoost (CAT) had the best performance in terms of predicting heart failure outcomes. It identified length of stay in the hospital, haemoglobin level, and family history of MI as the most important predictors for readmission, 1‐month mortality, and 1‐year mortality, respectively. These findings can thus be significant in helping doctors individualize HF patients at high risk of readmission or death.

We can therefore conclude that early detection of patients at risk of HF—through the use of ML—will allow for timely interventions to be made. Intensive monitoring of patients predicted to experience a negative outcome will help ensure the development of a more patient‐centered approach, forcing clinicians to ensure such patients are of utmost priority and tailor their treatment plans with caution and vigilance. The COACH trial found that using a tool in the emergency department to guide management plans for HF patients, combined with providing standardized transitional care, improved outcomes for such patients.^4^ This supports the idea that a ML‐based predictive model will aid in risk‐based decision‐making leading to better HF patient results, and also help reduce unnecessary hospital readmissions by identifying patients who require more attention post‐discharge. Additionally, doctors will be compelled to thoroughly counsel their patients, briefing them regarding specific lifestyle modifications required to lower the already very high risk of experiencing a similar cardiac event in the near future. Moreover, given the immense economic burden HF presents with, the use of ML will allow resources to be allocated efficiently based on predicted risk. In spite of all these merits, the current study has certain limitations including its retrospective design that used the records from the Fasa Registry on Systolic Heart Failure (FaRSH). For this reason, we recommend conducting prospective studies to overcome potential selection bias that may be associated with the study's current design. Moreover, the study population comprised mainly of patients of Arab and Persian ethnicity. Given the differences in incidence of congestive heart failure by ethnicity,^5^ this highlights the need for similar studies to be conducted on a wider geographical scale for more conclusive findings.

## AUTHOR CONTRIBUTIONS

Rayyan Nabi came up with the idea for the letter and drafted the manuscript. Tabeer Zahid helped in writing the manuscript and provided supportive ideas for its completion. Hanzala Ahmed Farooqi reviewed and supervised the article to ensure its clarity.

## CONFLICT OF INTEREST STATEMENT

The authors declare no conflict of interest.
